# Putative mechanisms of ocular inflammation in syphilis

**DOI:** 10.1186/s12348-026-00638-2

**Published:** 2026-09-15

**Authors:** Liam M. Ashander, João M. Furtado, Keryn A. Williams, Giles Best, Melissa H. Brown, Justine R. Smith

**Affiliations:** 1 Flinders Health and Medical Research Institute, College of Medicine and Public Health, Flinders University, Adelaide, Australia; 2 Ribeirão Preto Medical School, University of São Paulo, Ribeirão Preto, Brazil; 3 College of Science and Engineering, Flinders University, Adelaide, Australia; 4 ARC Training Centre for Biofilm Research and Innovation, Flinders University, Adelaide, Australia

**Keywords:** Syphilis, Ocular syphilis, Syphilitic uveitis, Retina, Infection, *Treponema pallidum*, Mechanisms

## Abstract

**Background:**

Ocular syphilis is re-emerging globally as a cause of uveitis, with the potential for substantial vision loss. Multimodal ophthalmic imaging and advanced laboratory research can yield important information about the basic mechanisms of this infectious eye disease.

**Review:**

Inflammation of the retina is a frequent manifestation of ocular syphilis. Observations from multimodal ophthalmic imaging suggest the retinal pigment epithelium and retinal vasculature are often involved. Molecular profiling of biopsies from patients with syphilis involving the eye and brain indicates multiple leucocyte subsets infiltrate the posterior eye, and highlights the role of monocytes and macrophages in promoting inflammation. Aqueous and cerebrospinal fluid samples from patients with ocular syphilis contain high levels of inflammatory mediators, including C-C motif chemokine ligand (CCL) 2, C-X-C motif chemokine ligand (CXCL) 8, interleukin (IL)-6, IL-12, and tumour necrosis factor (TNF). Macrophages and dendritic cells exposed to the causative bacterium, *Treponema pallidum*, increase the production of pro-inflammatory cytokines, such as IL-1β, IL-6, and TNF. Toll-like receptor activation and nuclear factor of kappa light chain enhancer of B-cells (NFκB) signalling in non-ocular human cells play prominent roles in upregulating inflammatory molecules, and altering interactions between leucocytes and epithelial or endothelial cells; similar cellular and molecular changes at the outer and inner blood-retinal barriers could contribute to retinal inflammation in ocular syphilis. Experimental evidence also indicates that *T. pallidum* alters intercellular junctions and transmigrates endothelial cell monolayers, potentially mediating retinal invasion.

**Conclusions:**

Evidence from multimodal ophthalmic imaging, molecular profiling in patients, and non-ocular cell infection models provides essential insights into the pathogenesis of ocular syphilis. This knowledge will target future research towards elucidating the drivers of retinal inflammation in ocular syphilis.

**Supplementary Information:**

The online version contains supplementary material available at https://doi.org/10.1186/s12348-026-00638-2.

## Background

The incidence of syphilis is rising across the globe: information collected in the Global Burden of Disease database indicates a surge from approximately 8 million patients in 1990 to 14 million in 2019 [1]. Along with this increasing case incidence, ocular syphilis is being encountered more frequently in ophthalmic practice [2]. Posterior segment intraocular inflammation is the most common presentation of ocular syphilis, and may result in irreversible vision impairment or blindness if antibiotic treatment is delayed [3, 4].

There has been limited basic research into the pathogenic mechanisms of ocular syphilis. In part, this reflects a decreasing incidence until recently, but technical issues have also been at play, including the challenges involved in culturing the causative bacterium, *Treponema pallidum*, and a lack of animal models that reliably recapitulate clinical features as observed in patients. Multimodal ophthalmic imaging provides important opportunities to understand the involvement of posterior segment tissue layers in the disease [3]. In addition, studies involving other body systems may be highly relevant. For example, immune crosstalk between the posterior eye and brain suggest the two may share similarities in infiltrating leucocyte populations and molecular profiles in response to *T. pallidum* infection [5]. Thus, the responses of extraocular epithelial and vascular endothelial cell populations to infection with *T. pallidum* may hold clues to how the retinal pigment epithelial cell and retinal endothelial cell barriers respond, and how exposure may shape their interactions with leucocytes.

This review article brings together multimodal ophthalmic imaging studies, and ocular and relevant non-ocular mechanistic investigations, to synthesise current knowledge of the putative mechanisms of ocular syphilis. Literature was sourced primarily by searching the National Institutes of Health National Library of Medicine PubMed database between May and October, 2025, using combinations of the following search terms: *syphilis*,* ocular syphilis*,* Treponema pallidum*,* eye*,* infection*. Additional literature was identified by review of reference lists and periodic updating of the original searches. The article begins with a short summary of the epidemiology, clinical features, and outcomes of ocular syphilis, as well as the microbiology and genomics of *T. pallidum*, and concludes with a brief discussion of potential future studies to advance understanding of this re-emerging infectious eye disease.

## Clinical and microbiological aspects

### Epidemiology of ocular syphilis

In a large international survey conducted in 2017, over 50% of uveitis-specialised ophthalmologists had observed an increase in cases of syphilitic uveitis over the past decade [2]. Consistently, recent population- and clinic-based studies from the Americas, Europe, Asia and Oceania have documented surging rates of ocular syphilis [6–11]. Ocular inflammation is an uncommon manifestation of syphilis, representing 0.6%-2.7% of all presentations [12–15]. Large case series indicate that a disproportionate number of patients with ocular syphilis are men, in particular men who have sex with men and men living with human immunodeficiency virus (HIV) infection [10, 16, 17]. The recent syphilis epidemic is not confined to these populations, however, as evidenced by reports of increased notifications amongst heterosexual men and women, and of congenital infections [18–20].

### Clinical presentation of ocular syphilis

The diversity of manifestations of syphilis has led to the common description, “the great masquerader”, and ocular syphilis is also well known to mimic a broad range of ophthalmic conditions [21–24]. One systematic review that included 362 patients with ocular syphilis showed that subjective complaints of vision loss or blurry vision were reported by approximately 90% of patients [25]. Observational studies from different countries indicate that the most common clinical presentation of ocular syphilis is panuveitis or posterior uveitis [10, 26–29]. Ocular involvement is frequently bilateral, diagnosed in between 47% and 69% of patients in these reports. A systematic review of 28 studies that described 670 patients with ocular syphilis identified optic nerve involvement (i.e., papillitis, optic neuritis, or neuroretinitis) to be another common manifestation [30].

### Outcomes of ocular syphilis

With timely diagnosis and treatment, the prognosis for ocular syphilis can be good; a large systematic review of 95 studies assessing visual acuity (VA) outcomes in patients with ocular syphilis demonstrated that final VA remained stable or was improved over baseline in 97% of affected eyes [25]. Where information on treatment was reported, intravenous penicillin G was usually prescribed, with some patients receiving intravenous ceftriaxone, in agreement with a recent practice pattern survey for syphilitic uveitis [2]. The authors found no significant association between HIV infection status and VA outcomes, although they did report an association between female sex and final LogMAR of greater than 1 [25]. In contrast, analysis of a Brazilian cohort of patients with ocular syphilis found no significant association between female gender and poor final VA [31]. Diagnostic and treatment delays, and presenting VA are significant prognostic factors for final VA outcomes in patients with ocular syphilis; for example, one retrospective study from Brazil indicated permanently reduced VA occurred in approximately 40% of 214 affected eyes, which the authors attributed to an average 3-month interval between the onset of symptoms and diagnosis [10]. Other large retrospective studies from The Netherlands and China have also highlighted the risk to vision of a delay in starting treatment [32, 33]. A retrospective chart review of 54 patients with syphilitic uveitis showed a correlation between longer duration of symptoms prior to treatment and the presence of cystoid macular oedema, and subsequent progression to chronic uveitis [34].

### Biology of *Treponema pallidum*

**Fig. 1:**
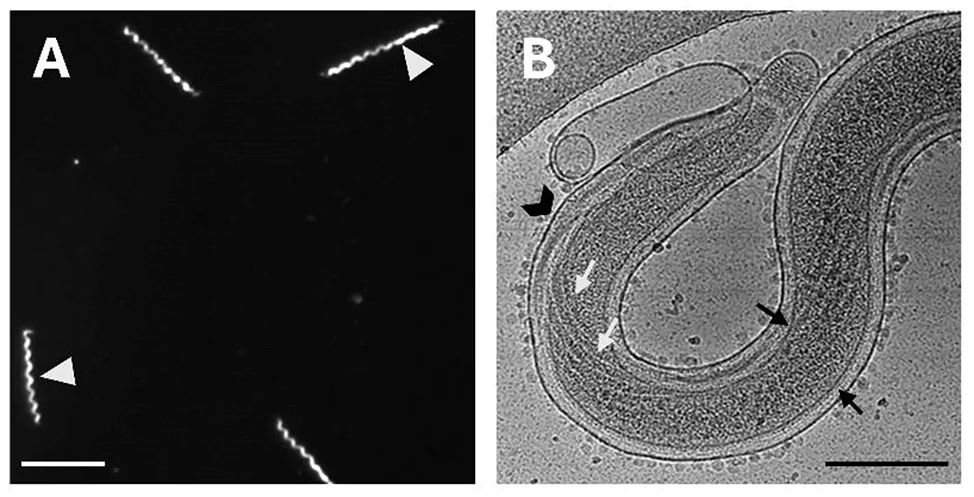
Spirochaete morphology of *Treponema pallidum*. Dark-field photomicrographs of Nichols strain *T. pallidum* (**A**). Original image magnification = 100X; scale bar = 10 μm. Cryo-electron microscopy of the spirochaete showing outer membrane (black arrowhead), inner cytoplasmic membrane (black arrows) and periplasmic flagella (white arrows) (**B**). Scale bar = 200 nm. Adaptation of original photomicrographs from Long-term in vitro culture of the syphilis spirochete *Treponema pallidum* subsp. *pallidum* © 2018 by D. G. Edmondson, B. Hu and S. J. Norris is licensed under Creative Commons Attribution 4.0 International [38]

The causative agent of syphilis, *Treponema pallidum* subsp. *pallidum*, is a Gram-negative spirochaete bacterium. Its cytoplasmic membrane is enclosed by a thin peptidoglycan sheath and a loose outer membrane which does not contain lipopolysaccharide (LPS) [35, 36]. This lack of LPS, coupled with the paucity of proteins in the outer membrane, may contribute to the ability of *T. pallidum* to evade the host immune system [37]. A periplasmic space between its two membranes contains the endoflagella that drive *T. pallidum*’s characteristic corkscrew motility [38] (Fig. 1) [39]. The genome of *T. pallidum* is small compared to that of other pathogenic bacteria, comprising 1.14 million base pairs encoding approximately 1,000 open reading frames [40].

The reduced genome reflects a high level of adaptation to living in a mammalian host and a limited metabolic capacity. Nevertheless, *T. pallidum* is highly invasive, able to move rapidly from the site of primary infection into the bloodstream, where it disseminates to other body tissues, including the central nervous system (CNS), by crossing endothelial barriers [41, 42]. The characterisation of an alternative metabolic pathway that generates ATP from the conversion of exogenous lactate to acetate, may partially explain how metabolically poor *T. pallidum* maintains the motility needed to rapidly disseminate in the mammalian host [43–46]. It is tempting to speculate that a sustained lactate gradient generated by the metabolic circuit between the retinal pigment epithelium and photoreceptors [47, 48], might contribute to the frequent involvement of the choriocapillaris-outer retina complex in ocular syphilis, although this hypothesis has not been explored experimentally to date.

### Clinical genomics of *Treponema pallidum*

Although there is a paucity of sequencing data from many regions of the world, *T. pallidum* is widely believed to exist in two dominant genetic lineages, the Nichols-like strains and Street strain 14 (SS14)-like strains, with no confirmed reports of mixed or intermediate genotypes [49, 50]. A large phylogenetics study, based on sequencing of 726 contemporary and historic *T. pallidum* isolates from 23 countries around the globe, revealed the striking finding that contemporary strains expanded rapidly following a population bottleneck in the 1990s and are genetically distinct from the strains in circulation prior to this time period [49]. Globally, SS14-like strains predominate in clinical samples, but recent reports have suggested that Nichols-like strains may be more widespread than previously thought [51, 52].

Genomic epidemiological studies from England, Australia, and Japan have indicated that distinct sub-lineages of *T. pallidum* are circulating amongst heterosexuals versus men who have sex with men, raising the possibility that oculotropic strains may be spread within discrete sexual networks [53–55]. In one case series from the US state of Michigan, authors reported that 5 women developed ocular symptoms after contracting syphilis from a common male sex partner, although *T. pallidum* genetic material could not be isolated for genotyping [56]. Following a US Centers for Disease Control report of an ocular syphilis cluster in 2014–2015, multi-locus sequence typing of ocular and non-ocular isolates from 14 patients revealed no predominant strain; however, an unusual strain genotype, rare in the US, was identified in one patient and suspected in their partner, both with ocular syphilis [57]. A more recent investigation also found no genotype was over-represented in isolates from patients with ocular syphilis, with complete genotypes determined in 6 of 17 isolates [58].

### Antibiotic resistance in *Treponema pallidum*

Resistance of *T. pallidum* to macrolide antibiotics has been well documented for clinical isolates, with many reports confirming that greater than 75% harbour resistance-conferring single-nucleotide polymorphism (SNPs) A2058G or A2059G in the 23s ribosomal DNA [59–62]. In studying 604 *T. pallidum* isolates from the US and Canada, investigators found 99.2% to be genotypically macrolide-resistant [63]. Several case reports and case series have documented treatment failures in patients with syphilis treated with macrolide antibiotics [64–67]. A recent in vitro mutagenesis study indicated that SNPs present in a *T. pallidum* isolate, collected from a patient whose infection failed treatment with ceftriaxone, could confer partial resistance to penicillin G [68]; however, subsequent work with 3 clinical isolates naturally harbouring these SNPs demonstrated they were not less susceptible to bactericidal concentrations of ceftriaxone or penicillin G [69]. A multi-centre case-control study is currently underway to determine the relationship between mutations in *T. pallidum* penicillin-binding genes and reduction of Venereal Disease Reference Laboratory (VDRL) or Rapid Plasma Reagin (RPR) titres following penicillin G or procaine penicillin treatment [70]. The available data indicate that while macrolide antibiotics are frequently ineffective against *T. pallidum* due to widespread resistance, resistance of the bacterium to penicillin G and ceftriaxone is not a pressing clinical issue currently.

## Multimodal ophthalmic imaging in ocular syphilis

### Optical coherence tomography

**Fig. 2:**
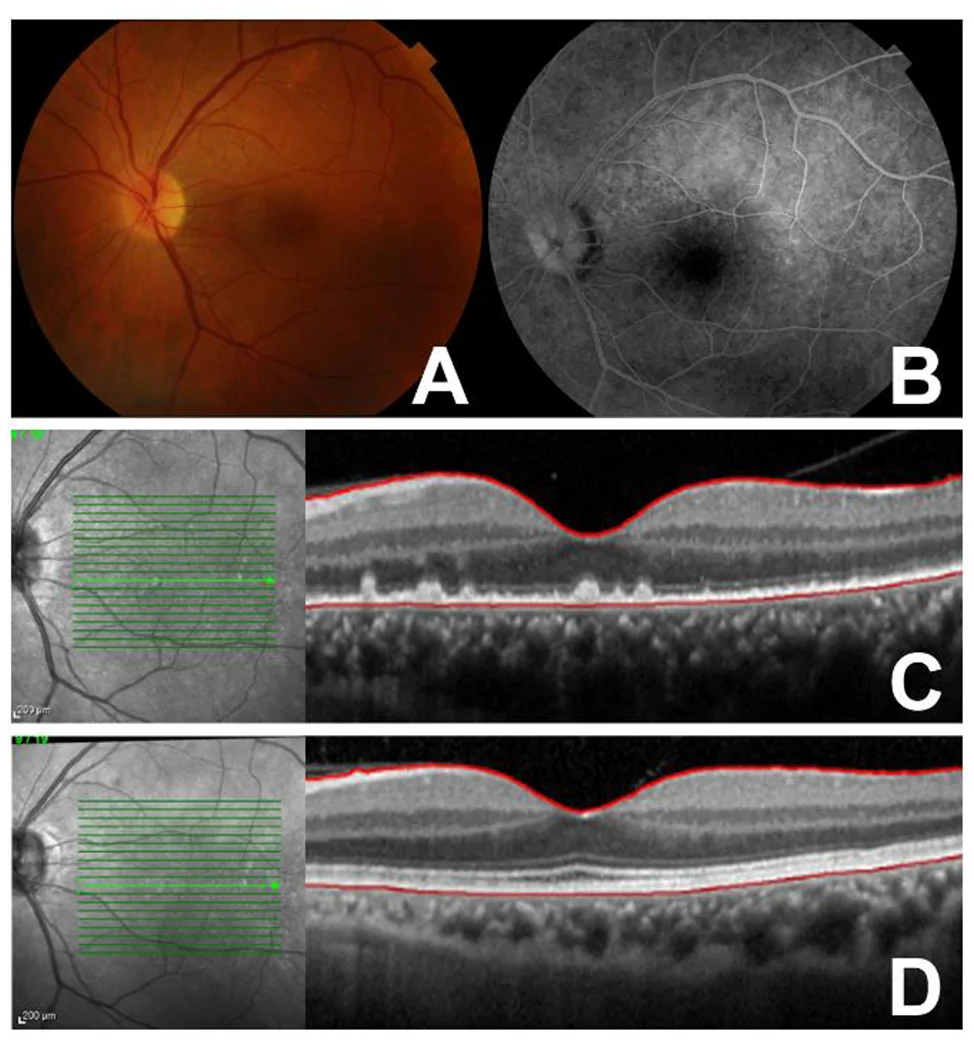
Ocular syphilis with placoid outer retinal involvement. Colour fundus photograph shows subtle yellow-white placoid changes involving the posterior pole, with mild optic disc hyperaemia (**A**). Fluorescein angiography demonstrates granular hyperfluorescence in the affected area, compatible with active outer retinal and retinal pigment epithelial involvement (**B**). Optical coherence tomography shows disruption and irregularity of the outer retinal bands and retinal pigment epithelium in the affected area (**C**), with substantial improvement after appropriate antibiotic treatment (**D**). Informed consent was obtained from the patient as an enrolled subject of an observational study of ocular syphilis that included ocular imaging

Several reports have detailed optical coherence tomography (OCT) features that support involvement of the retinal pigment epithelium in ocular syphilis, and specifically syphilitic uveitis (Fig. 2) [71–74]. In a single-centre study of 54 Brazilian patients with ocular syphilis, disruption of the ellipsoid zone (EZ) was observed in 61% of 54 eyes, and hyper-reflective nodular elevations or other retinal pigment epithelium changes were seen in between 59% and 70% [73].

In patients followed for at least 14 weeks after treatment with intravenous penicillin G or intravenous ceftriaxone, EZ changes persisted in 51% of 31 affected eyes, while the retinal pigment epithelium remained thickened or irregular in almost 40%. There was a trend towards worse final VA in eyes presenting with EZ disruption. Hyper-reflective nodular retinal pigment epithelium elevations were described in 45% of eyes from 40 patients with ocular syphilis in an independent study [71]. In a case series of 24 eyes from 17 patients with acute syphilitic posterior placoid chorioretinitis (ASPPC) from a single centre in the US state of Michigan, the authors reported EZ disruption in 83% of eyes and focal hyper-reflectivity of the retinal pigment epithelium in 75% at presentation [75]. There was persistent EZ disruption in over 40% of eyes after treatment with intravenous penicillin G, and a trend towards worse final VA in eyes presenting with foveal EZ disruption.

### Optical coherence tomography angiography

Blood flow assessment in the retinal capillary plexuses and choroidal capillaries by OCT angiography in patients with syphilitic outer retinitis or ASPPC has shown flow signal voids underlying retinal lesions, which largely resolve with antibiotic treatment; the authors of these reports attributed the findings to local ischaemia or inflammation [76–79]. There are descriptions of patients presenting with unilateral ASPPC, but with hyper-reflective choroidal dots on OCT and choroidal flow signal voids on OCT angiography in the fellow eye, leading investigators to propose subtle involvement of the choroid before the development of retinal pathology [80]. The morphological changes in the retinal pigment epithelium and choroid observed during syphilitic uveitis suggest the retinal pigment epithelium is involved in the pathogenesis of this condition.

### Fundus autofluorescence

**Fig. 3:**
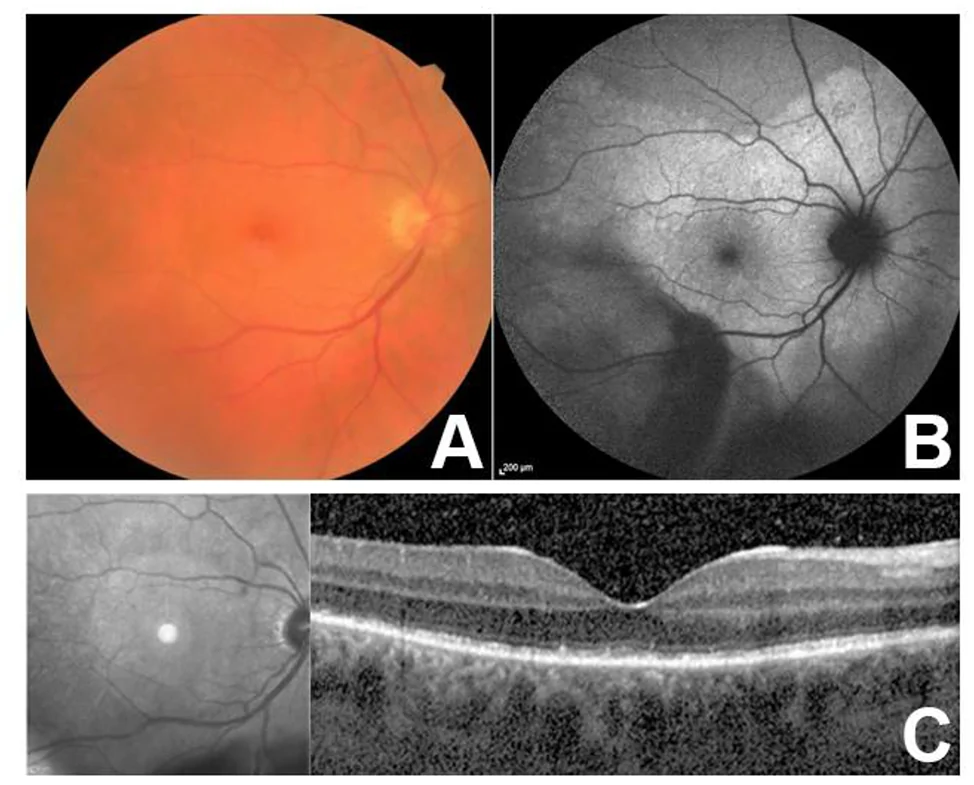
Subtle outer retinal involvement in ocular syphilis. Colour fundus photograph shows mild posterior pole changes with limited clinically visible retinal abnormalities in part due to vitritis (**A**). Fundus autofluorescence highlights a placoid area of altered signal involving the posterior pole, more evident than on colour photography (**B**). Optical coherence tomography through the macula shows preservation of the foveal contour with subtle outer retinal and retinal pigment epithelial abnormalities (**C**). Informed consent was obtained from the patient as an enrolled subject of an observational study of ocular syphilis that included ocular imaging

Fundus autofluorescence (FAF) imaging in a series of 3 patients with syphilitic outer retinitis highlighted hyperautofluorescence of the retinal lesions in all, corresponding to areas of EZ disruption on OCT (Fig. 3) [81]. In 2 patients, the authors observed a sharp transition from hyperautofluorescence to normal autofluorescence, which corresponded to the limit of EZ zone disruption in the lesion on OCT and improved substantially within 3 months of treatment with intravenous penicillin G. The authors attributed the hyperautofluorescence to loss of photopigments in the photoreceptors, while acknowledging that accumulation of lipofuscin was also possible. In another report of 4 eyes of 3 patients with ASPPC, FAF imaging showed overall hyperautofluorescence of the lesions, with bright punctate areas corresponding to nodular elevations of the retinal pigment epithelium by OCT [82]. The authors of this work suggested that the alterations on the FAF might reflect accumulation of lipofuscin or photoreceptor outer segments in a dysfunctional retinal pigment epithelium.

### Fluorescein and indocyanine green dye angiography

**Fig. 4:**
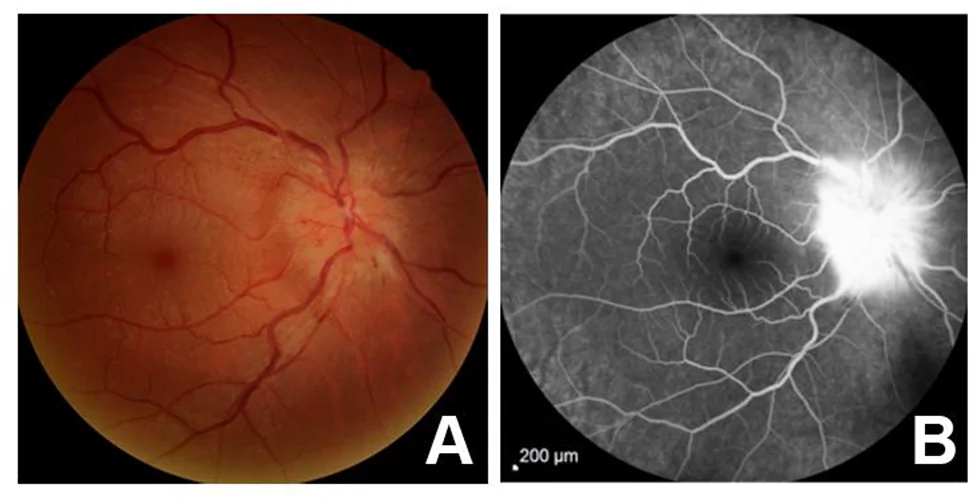
Syphilitic papillitis and neuroretinitis. Colour fundus photograph shows optic disc oedema with peripapillary retinal whitening and vascular congestion (**A**). Fluorescein angiography demonstrates marked optic disc leakage, consistent with active inflammation and optic disc involvement (**B**). Informed consent was obtained from the patient as an enrolled subject of an observational study of ocular syphilis that included ocular imaging

Fluorescein and indocyanine green dye angiographic studies indicate that alterations of retinal vascular barrier integrity and choroidal perfusion are common in ocular syphilis. A retrospective review of fluorescein angiographic examinations from 23 patients with ocular syphilis revealed staining of retinal vessels and optic disc hyperfluorescence in 30% of eyes, implying increased permeability of the retinal vascular endothelium (Fig. 4) [83]. In the same study, the authors reported hypocyanescent areas in the choroid of 59% of eyes by indocyanine green angiography, and proposed this was indicative of choroiditis. Other small case series using indocyanine green angiography demonstrated progressive hypocyanescence in the choroid underlying retinal lesions; some authors speculated the hypocyanescence was caused by reduced choroidal perfusion associated with inflammation, whereas others interpreted the change as retinal pigment epithelial cell dysfunction [84–86].

### Summary

Dye and OCT angiographic studies in patients with ocular syphilis indicate that *T. pallidum* infection affects both the retinal and choroidal circulations: breakdown of the inner blood-retinal barrier (BRB), outer retinal ischaemia, and associated choroidal changes all may contribute to the observed pathology. The FAF and OCT findings in ocular syphilis imply that the disease is accompanied by alterations in retinal pigment epithelial cell morphology. Abnormalities of the retinal pigment epithelium and disruption of the EZ can persist after treatment, and may contribute to worse final VA. However, imaging alone often cannot establish the mechanisms responsible for these morphological changes. The cellular and molecular mechanisms underlying ocular syphilis may be further informed by studies involving patients with concurrent ocular and CNS infections, as well as in vitro mechanistic studies (Supplementary Table 1).

## Relevant cellular and molecular observations

### Outer blood-retinal barrier

The immunological and metabolic micro-environments of the posterior eye are maintained by the inner and outer BRBs, specialised barriers that regulate the passage of fluid, macromolecules, and leucocytes between the retina and the circulation. Along with Bruch’s membrane, the retinal pigment epithelial cells constitute the outer BRB [87]. Bruch’s membrane and the retinal pigment epithelial basement membrane form a multi-layered extracellular matrix (ECM) composed of collagen, elastin, laminin, and fibronectin isoforms [87]. Among other functions, this membranous structure provides physical support for the retinal pigment epithelium and contributes to the avascularity and fluid homeostasis of the outer retina. Key to the maintenance of the outer BRB is the formation of tight junctions between retinal pigment epithelial cells. Claudin-19 is the predominant tight junction protein in the retinal pigment epithelium [88]. Extracellular domains of claudin-19 and structurally similar occludin (OCCL) form cell-cell junctions, while their cytoplasmic domains interact with the actin cytoskeleton via the zonula occludens (ZO)-1 scaffolding protein [89]. Junctional adhesion molecule (JAM)-C likely plays a role in tight junction assembly in the retinal pigment epithelium by regulating ZO-1 and cadherin localisation [90].

### Immunomodulatory activity of the retinal pigment epithelium

Beyond the physical barrier formed by tight junctions, retinal pigment epithelial cells express a wide variety of cytokines, chemokines, growth factors, co-stimulatory molecules, and cell adhesion molecules that may profoundly influence the ocular immune response [91]. Retinal pigment epithelial cells produce immunoregulatory factors such as alpha melanocyte-stimulating hormone [92], pigment epithelium derived factor [93], thrombospondin, transforming growth factor beta [94], and interleukin (IL)-1 receptor agonist [95] that generate regulatory T-cells which can supress pro-inflammatory lymphocyte activity [96]. Similarly, retinal pigment epithelial cells express immunoregulatory molecules such as Fas ligand, programmed cell death 1 ligand 1 (PD-L1), and PD-L2 on the cell surface, which can regulate the activation state of multiple leucocyte subsets [97–99]. On the other hand, under disease conditions, retinal pigment epithelial cells may exacerbate inflammation and retinal damage by up-regulating pro-inflammatory cytokines and chemokines that recruit and activate leucocytes, cell adhesion molecules that mediate leucocyte extravasation, and metalloproteinases that alter the structure of the ECM [100–102]. Thus, the activity of the retinal pigment epithelium is a crucial balance point for the ocular immune response, potentially reducing retinal damage by regulating leucocyte behaviour or exacerbating inflammation by contributing to leucocyte recruitment and activation. Because the retinal pigment epithelium shuttles glucose into the subretinal space to support photoreceptor metabolism [103], any disruption of the outer BRB also may result in metabolic stress and mitochondrial dysfunction in photoreceptors.

### Inner blood-retinal barrier

The inner BRB is maintained as part of a neurovascular unit that couples vascular responses and neuronal activity to ensure adequate nutrient supply and waste removal in physiologically active areas of the retina [104]. Retinal capillaries are ensheathed by cells that include pericytes and Müller glial cells, which support and maintain the vascular endothelial monolayers and basement membrane [89]. Pericyte loss and subsequent retinal endothelial cell drop-out contributes to breakdown of the inner BRB [105]. A critical component of the inner BRB is the formation of tight junctions between the endothelial cells that line the retinal blood vessels [106]. Claudin-5 is a key protein for tight junction formation in the retinal endothelium [107], in addition to OCCL, JAMs, and ZOs. The adherens junction protein, vascular endothelial (VE)-cadherin, is also important for stabilizing the inner BRB; disruption of VE-cadherin protein integrity or localization leads to increased retinal vascular permeability [108, 109].

### Leucocyte infiltration of the choroid and retina in ocular syphilis

Data on the leucocyte populations infiltrating the retina and choroid during ocular syphilis are sparse, although a few historic histopathological studies exist. In a case report describing post-mortem histology of the left eye of an infant congenitally infected with syphilis, a dense necrotic area of the iris was surrounded by granulocytes, macrophages, and lymphocytes, and spirochaetes were detected by Krajian’s silver stain in the lens [110]. The authors noted similar, but less intense, inflammation in the retina and choroid. In another case report, in which Krajian’s silver stain-positive spirochaetes were present throughout the enucleated eye of a 58-year-old patient with congenital syphilis, the retina and choroid were infiltrated with multi-nucleated giant cells or granulomas [111]. A case series reporting the histopathology of retinochoroidal biopsies from 23 patients with posterior uveitis included the results of a biopsy from one patient with syphilitic retinitis [112]. This biopsy was characterised by a macrophage-dominated infiltrate in the choroid and retina, along with granulomas. Except for CMV retinitis, all other forms of uveitis in this case series were characterised by predominantly T-cell or B-cell infiltration. These studies suggest infiltration of the retina and choroid by a mixed leucocyte population, and a prominent role for macrophages in ocular syphilis.

### A shared immune response in neurosyphilis and ocular syphilis?

Approximately 70% of patients with ocular syphilis have abnormal cerebrospinal fluid (CSF) findings [28, 113, 114], although the interpretation of published studies is complicated by variation in practice related to indications for lumbar puncture, analysis of CSF, diagnostic criteria for neurosyphilis and ocular syphilis, as well as HIV infection status of the subjects. A recent publication provides evidence of a discrete lymphatic circuit shared between the posterior eye and brain in mouse models of viral and bacterial CNS infections [115]. This suggests that examining the immune response in neurosyphilis might yield information about those occurring inside the eye, recognising that anterior chamber, vitreous cavity, retina, choroid, and subarachnoid space are not equivalent compartments structurally or immunologically. Flow cytometric analysis of CSF from 6 patients with neurosyphilis indicated that the average infiltrating lymphocyte population was 54% CD4 + T-cells, 19% CD8 + T-cells, and 20% B-cells [116]. A single-cell transcriptomic profiling study of CSF from 3 patients with neurosyphilis produced similar results for T-cells and B-cells, but a higher proportion of monocytes and plasma cells [117]. This study identified a strong Toll-like receptor (TLR)- nuclear factor of kappa light chain enhancer of B-cells (NFκB) transcriptomic signature in brain tissue from 3 autopsy cases of neurosyphilis, with up-regulation of CD14, IL-12, and tumour necrosis factor (TNF), as well as downstream TNF-receptor associated factors (TRAF) 2, TRAF3 and TRAF6.

### Cytokine profiles in neurosyphilis and ocular syphilis

One profiling study of differentially expressed soluble factors in the CSF of 112 patients with various manifestations of syphilis, which included 9 patients with ocular syphilis, indicated that CSF profiles of patients with ocular syphilis clustered with asymptomatic neurosyphilis and latent syphilis [118]. The cluster was defined by predominantly Th_1_ cytokines, including IL-2, IL-12p70, interferon (IFN)-γ, and TNF. Another profiling study compared cytokine levels in aqueous and plasma from 16 HIV-positive patients with ocular syphilis, reporting higher levels of granulocyte colony-stimulating factor (G-CSF), granulocyte-macrophage colony-stimulating factor (GM-CSF), IL-1α, IL-6, C-C motif chemokine ligand (CCL) 2, CCL3, C-X-C motif chemokine ligand (CXCL) 8, and CXCL10 in aqueous [119]. A third study of 23 patients with ocular syphilis found elevated levels of CCL4, IL-2, IL-10, and IL-12p70 in the aqueous compared to the CSF, regardless of neurosyphilis or HIV infection status [120]. Interestingly, the concentration of IFN-γ in aqueous was significantly reduced in patients with neurosyphilis compared to those with no CSF abnormalities. The potential disease involvements of soluble factors detected in the aqueous during ocular syphilis are presented in Table 1.

**Table 1:** Soluble factors detected in aqueous of patients with ocular syphilis and potential involvements in the disease pathogenesis

Molecule [References]	Intraocular source	Potential involvement(s) in pathogenesis
CCL2 [119]	Iris pigment epithelial cells [121] Müller cells [122, 123] Microglial cells [124–126] Retina endothelial cells [127, 128] Retinal pigment epithelial cells [127, 129]	**Inflammation** Intravitreal CCL2 injection increased monocyte/macrophage recruitment into mouse retina [130] CCL2 siRNA knockdown reduced macrophage/microglial cell recruitment in rat retina following light damage [122] CCL2 knockout reduced decreased monocyte/macrophage infiltration and ganglion cell death in a mouse model of glaucoma [131] CCL2 knockout reduced inflammation in mouse retina during bacterial endophthalmitis [132]
		**Barrier dysfunction** CCL2 altered OCCL, ZO-1 and claudin-5 localisation in mouse brain endothelial cells [133, 134] CCL2 siRNA knockdown restored OCCL and ZO-1 expression and decreased barrier permeability in ARPE-19/microglial cell co-culture [135]
CCL3 [119]	Microglial cells [136, 137] Retinal pigment epithelial cells [138]	**Inflammation** CCL3 knockout reduced inflammation in mouse retina during bacterial endophthalmitis [132] CCL3 knockout attenuated light-induced damage in Stargardt Disease mouse model [136] Anti-CCL3 treatment reduced leucocyte adherence and extravasation in experimental autoimmune uveitis mouse model [139] **Barrier dysfunction** Anti-CCL3 treatment reduced Evans Blue leakage from retinal vessels in mouse experimental autoimmune uveitis [139]
CCL4 [119, 120]	Iris pigment epithelial cells [121] Microglial cells [137] Retinal endothelial cells [140] Retinal pigment epithelial cells [138]	**Inflammation** Anti-CCR5 treatment reduced T-cell recruitment in mouse experimental autoimmune uveitis [141] Anti-CCL4 treatment reduced monocyte infiltration in mouse oxygen-induced retinopathy [142] **Barrier dysfunction** CCL4 treatment altered ZO-1 and VE-cadherin localisation and increased dextran permeability in HCMEC/D3 cells [143]
CXCL8 [119, 144]	Iris pigment epithelial cells [121] Müller cells [126, 145, 146] Retinal endothelial cells [145] Retinal pigment epithelial cells [101, 126, 147]	**Inflammation** CXCL8 induced primary human neutrophil migration across retinal endothelial cell monolayer in vitro [101] Correlation between vitreous CXCL8 concentration and CD45 + cells in fibrovascular membrane from patients with diabetic retinopathy [148]
		**Barrier dysfunction** CXCL8 treatment altered OCCL, ZO-1 and claudin-5 expression and increased monolayer permeability in EA.hy926 cells [149] CXCL8 treatment increased barrier permeability by VEGFR transactivation in hMVEC [150] Anti-CXCL8 treatment attenuated altered ZO-1 localisation and decreased barrier permeability in ARPE-19 cells treated with THP-1 cell supernatant [151] CXCL8 treatment induced death in ARPE-19 cells via CXCR1 signalling [152]
CXCL10 [119, 144]	Iris pigment epithelial cells [121] Müller cells [126, 153] Retinal endothelial cells [140, 154] Retinal pigment epithelial cells [126]	**Inflammation** Anti-CXCL10 treatment reduced PBMC adhesion to TNF-treated hRMEC monolayers [154] Anti-CXCL10 treatment decreased T-cells and IFN-γ expression in the mouse retina after *Toxoplasma gondii* infection [155] CXCL10 knockout reduced inflammation in mouse retina during bacterial endophthalmitis [156] Pathological astrocyte activation by CXCL10 increased complement-mediated retinal ganglion cell death [157]
CXCL13 [144]	Retinal pigment epithelial cells [158, 159]	**Inflammation** CXCL13 increased primary B-cell migration across human retinal endothelial cell monolayers in vitro [158] CXCL13 + tertiary lymphoid tissue formed in mouse retina during experimental autoimmune uveitis [160]
G-CSF [119]	Müller cells [161] Retinal pigment epithelial cells [101]	**Inflammation ** Intravitreal G-CSF injection reduced microglia activation, macrophage recruitment and ganglion cell apoptosis in a rat optic nerve crush model [162] Systemic G-CSF treatment reduced ganglion cell apoptosis and reduced TNF and IL-1β production in optic nerve tissues in a rat optic nerve ischaemia model [163] **Barrier function** G-CSF treatment reduced ROS-induced apoptosis in a human retinal endothelial cell line [164]
GM-CSF [119]	Müller cells [161] Retinal pigment epithelial cells [101]	**Inflammation** GM-CSF is a key contributor to pathogenic Th17 autoimmunity [165] Overexpression of GM-CSF in mouse retinal explants increased microglial cell activation and expression of *ccl2* and *cxcl10* transcripts [166] GM-CSF-mediated eosinophil recruitment enhanced retinal inflammation in a mouse experimental autoimmune uveitis [167]
IL-1α [119]	Retinal pigment epithelial cells [101, 168, 169] Microglial cells [170, 171]	**Inflammation** IL-1α treatment increased production of IL-6, CCL2 and CXCL8 by ARPE-19 cells [168] IL-1α priming increased inflammasome activation, IL-1β/IL-18 production and pyroptotic cell death in primary retinal pigment epithelial cells and ARPE-19 cells [172–174] Macrophage IL-1α induced pathological astrocyte activation and ganglion cell death in a mouse glaucoma model [175]
IL-6 [119, 120]	Iris pigment epithelial cells [121] Microglial cells [126, 176] Müller cells [126] Retinal pigment epithelial cells [101, 126]	**Inflammation** Il-6 treatment activated NFκB signalling and increased TGFβ2 production in ARPE-19 cells [177] IL-6 increased macrophage/microglial cell recruitment to the retinal pigment epithelium in mouse retina and up-regulated TNF production by microglial cells in vitro [178] IL-6/sIL-6R treatment increased ROS production and ICAM-1 expression in human retinal endothelial cells [179]
		**Barrier dysfunction** IL-6 treatment increased monolayer permeability and altered junctional molecule localisation in ARPE-19 cells [180] Human retinal endothelial cells express membrane-bound IL-6R; IL-6 treatment increased human retinal endothelial cell barrier permeability [181] IL-6/sIL-6R treatment increased apoptosis and endothelial barrier permeability in human retinal endothelial cells [179] IL-6 treatment reduced expression of OCCL and ZO-1 via autocrine VEGF signalling in human retinal endothelial cells [176]
IL-10 [120]	Amacrine cells [182] Microglia [183] Müller cells [184] Photoreceptors [184]	**Inflammation ** IL-10 knockout increased retinal necrosis in a mouse model of ocular toxoplasmosis [185] IL-10 knockout increased leucocyte infiltration and retinal pathology in a mouse model of autoimmune retinopathy [186] STAT-1 knockout impaired IL-10 production and increased retinal pathology in mouse autoimmune uveitis [184] IL-10 treatment reduced IL-1β and IL-6 production by rat retinal pigment epithelial cells [187]
		**Barrier dysfunction** IL-10 siRNA knockdown in M2 macrophages decreased ZO-1 and OCCL expression and increased barrier permeability in an ARPE-19 cell co-culture model [188] IL-10 treatment reduced rat retinal pigment epithelial cell proliferation and migration [187]

Abbreviations: CCL = c-c motif chemokine ligand; CCR = c-c motif chemokine receptor; CXCL = c-x-c motif chemokine ligand; CXCR = c-x-c motif chemokine receptor; G-CSF = granulocyte colony stimulating factor; GM-CSF = granulocyte macrophage colony stimulating factor; hCMEC/D3 = human cerebral microvascular endothelial cell d3; hMVEC = human dermal microvascular endothelial cell; IFN = interferon; IL = interleukin; OCCL = occludin; PBMC = peripheral blood mononuclear cells; ROS = reactive oxygen species; siRNA = small interfering RNA; STAT1 = signal transducer and activator of transcription 1; TGFβ = transforming growth factor beta; TNF = tumour necrosis factor; VEGF = vascular endothelial growth factor; ZO-1 = zonula occludens 1

### CXCL13 in ocular syphilis

CXCL13, a B-cell chemokine, has been proposed as a candidate biomarker for neurosyphilis [189–191]. A mechanistic study indicated CXCL13 may be key for recruiting B-cells into the CSF, and further, described “ectopic germinal centres” in the brain biopsy of a patient with tertiary syphilis [192]. Similar tertiary lymphoid structures, which labelled prominently for CXCL13, have been observed in the mouse retina in an experimental autoimmune uveitis model [160]. A recent study reported levels of CXCL8, CXCL10, and CXCL13 were significantly higher in aqueous compared to serum from patients with ocular syphilis [144]. Although its role in lymphocyte recruitment during ocular syphilis remains to be determined, CXCL13 has been reported to increase transmigration of B-cells across simulated retinal endothelial cell monolayers [158]. While not typically assessed in ocular syphilis, occasional case reports that included Goldmann-Witmer coefficients for antibodies against treponemal proteins have indicated possible intraocular antibody production, suggesting potential infiltration of B-cells or plasma cells into the eye [193, 194].

### Barrier cell responses to *Treponema pallidum*

At the time of writing, there are no published studies examining the effect of *T. pallidum* exposure on the retinal pigment epithelial cell host response. However, single-cell transcriptomic profiling of subsets of keratinocytes, another specialised epithelial cell, in skin lesion biopsies from 4 patients with secondary syphilis has revealed substantial dysregulation of epithelial cell differentiation and epithelial cell-cell adhesion [195]. This study also reported myeloid differentiation primary response 88 (MYD88)-dependent up-regulation of *CXCL1*, *CXCL8*, and *IL1B* transcripts in keratinocytes in response to live or sonicated *T. pallidum*. Stimulation of the HaCaT human keratinocyte cell line with recombinant *T. pallidum* flagellar proteins led to activation of mitogen-activated protein kinase (MAPK) and NFκB signalling via TLR 5 and MYD88, and increased the expression of matrix metalloproteinase (MMP) 9 and MMP13 [196]. In these same cells, stimulation with recombinant *T. pallidum* flagellar sheath protein also increased expression of IL-6 and CXCL8 protein via TLR2- and MYD88- dependent activation of NFκB and p38 MAPK signalling [197]. These studies suggest that exposure of the retinal pigment epithelium to *T. pallidum* could substantially dysregulate signalling pathways that are crucial for outer BRB integrity, and promote retinal damage by activating pro-inflammatory signalling pathways.

In a study that involved exposing HCMEC/D3 human cerebral microvascular endothelial cells to Nichols strain *T. pallidum*, there was increased secretion of IL-6, CXCL8, and vascular endothelial growth factor (VEGF) [198]. This study also reported decreased secretion of CCL2 protein by these same cells. In contrast, CCL2 protein up-regulation was reported in human dermal vascular smooth muscle cells and human umbilical vein endothelial cells (HUVEC) after incubation with Nichols strain *T. pallidum* and *T. pallidum* proteins Tp17 and Tp0965, respectively [199–201]. Activation of TLR2 by recombinant Tp0768 lipoprotein in HUVEC induced secretion of CXCL1, CXCL2, and CXCL8, and increased migration of primary neutrophils in a dual chamber transwell assay [202]. Molecular signature enrichment analysis of transcriptomic data from HCMEC/D3 endothelial cells exposed to Nichols strain *T. pallidum* showed enrichment of transcripts involved in epithelial-to-mesenchymal transition, apoptosis, the hypoxia response, and TNF signalling via NFκB [203]. These results provide further evidence that *T. pallidum* may activate barrier cells; in ocular syphilis this could contribute to inner BRB breakdown and uncontrolled leucocyte infiltration.

### Adhesion of *Treponema pallidum* to host cells

The Bruch’s membrane and retinal pigment epithelial basement membrane complex must be navigated by microorganisms or leucocytes breaching the outer BRB [204]. Several independent investigations undertaken with non-ocular cells and purified ECM components have identified candidate *T. pallidum* adhesins and ligands: Tp0751 which binds laminin and the laminin receptor; fibronectin-binding proteins Tp0136, Tp0155 and Tp0483; and Tp0954 which binds the glycosaminoglycans heparan and dermatan sulphate [205–209]. Preliminary evidence from heterologous expression of *T. pallidum* repeat protein K (TprK) in another spirochaetal pathogen, *Borrelia burgdorferi*, suggests it also may function as an adhesin [210]. Putative interactions between *T. pallidum* adhesins and host ECM components or receptors are presented in Table 2.

**Table 2:** *Treponema pallidum*-host cell binding proteins and ligands

*T. pallidum* protein	Ligand(s)	Binding assessed using	Major binding interactions	Additional observations	Reference
Tp0136	Fibronectin Laminin	Purified ECM components	Recombinant Tp0136 bound to fibronectin and laminin-coated plates	Bound weakly to laminin	[206]
	Fibronectin Laminin	Purified ECM components	Significantly increased Tp0136 binding to fibronectin and laminin-coated plates	Tp0136 bound both plasma and cellular fibronectin	[211, 212]
		Mammalian cells	Tp0136 expression in *B. burgdorferi* increased binding to mammalian cells: BeWo, C6, EA.Hy926, HEK293, Vero	Bound weakly to HEp-2 cells	[211]
Tp0155	Fibronectin	Purified ECM components	Significantly increased Tp0155 binding to matrix fibronectin		[209]
		Mammalian cells	Recombinant Tp0155 coating significantly increased SW480 cell attachment	AtT20 cells showed lower attachment	[209]
Tp0435		Mammalian cells	Tp0435 expression in *B. burgdorferi* increased binding to mammalian cells: C6, HEK293	Evidence of phase variation in Tp0435 expression	[213]
Tp0483	Fibronectin	Purified ECM components	Significantly increased Tp0483 binding to soluble and matrix fibronectin		[209]
		Mammalian cells	Recombinant Tp0483 coating significantly increased SW480 cell attachment	AtT20 cells showed lower attachment	[209]
Tp0751	Laminin	Purified ECM components	Recombinant Tp0751 bound to various laminin isoforms		[214]
		Mammalian cells	Recombinant Tp0751 coating significantly increased SW480 cell attachment		[215]
	Fibrinogen	Purified ECM components	Recombinant Tp0751 bound to fibrinogen-coated plates	Evidence Tp0751 also degraded laminin and fibrinogen	[216]
	Fibronectin Fibrinogen Collagen IV	Purified ECM components	Synthetic Tp0751 peptide fragments bound to fibrinogen, fibronectin and collagen-IV coated plates		[217]
	Laminin receptor	Mammalian cells	Recombinant Tp0751 bound to microvascular endothelial cells: HCMEC/D3, HMVEC, HUVEC	Evidence of LAMR-Tp0751 interaction by affinity chromatography and co-immunoprecipitation in HCMEC/D3	[207]
Tp0954	Dermatan sulfate Heparan sulfate	Mammalian cells	Tp0954 expression in *B. burgdorferi* increased binding to mammalian cells: BeWo, C6, EA.Hy926, HEK293, Vero	*B. burgdorferi* expressing Tp0954 bound to placental tissue sections	[208]
TprK		Mammalian cells	TprK expression in *B. burgdorferi* increased binding to mammalian cells: C6, HEK293, HeLa		[210]

Abbreviations: AtT20 = mouse pituitary tumour cell; BeWo = human choriocarcinoma cell; C6 = rat glioma cell; EA.Hy926 = human somatic hybrid cell; ECM = extracellular matrix; HCMEC/D3 = human cerebral microvascular endothelial cell d3; HEK293 = human embryonic kidney cell; HMVEC = human dermal microvascular endothelial cell; HEp-2 = human epithelial carcinoma cell; HUVEC = human umbilical vein endothelial cell; LAMR = laminin receptor; SW480 = human colon adenocarcinoma cell; Vero = African green monkey kidney epithelial cell

A single study has implicated extracellular matrix protein decorin in *T. pallidum* attachment to human brain microvascular endothelial cells; however, decorin-binding proteins homologous to those found in *Borrelia burgdorferi* have not yet been reported for *T. pallidum* [218]. In a gene expression microarray study of immortalised human brain microvascular endothelial cells co-incubated with Nichols strain *T. pallidum* spirochaetes, the transcript encoding decorin, *DCN*, was among the ten most highly up-regulated [219].

*T. pallidum* adhesin Tp0751 is co-transcribed and associates with Tp0750, a structurally related protein with proteolytic activity that cleaves MMP substrates and degrades fibrinogen and fibronectin [220]. Heterologous expression of Tp0136 in a non-adherent *B. burgdorferi* strain increased binding to the HEK293 human kidney epithelial cell line, and treatment with recombinant Tp0136 increased expression of MMP9 and altered the ratio of MMP9 to metalloproteinase inhibitor TIMP1 in human dermal vascular smooth muscle cells [211, 221]. These results suggest *T. pallidum* adhesins can mediate bacterial attachment to epithelial and endothelial cells, as well as breakdown of the extracellular matrix, which would facilitate invasion of the retina in ocular syphilis.

### Transmigration of cell barriers by *Treponema pallidum*

*T. pallidum* spirochaetes may penetrate the inner and/or outer BRB to access the posterior eye. Transmigration of cell barriers by *T. pallidum* has been investigated in vitro with human endothelial cell monolayers, although no published studies have used human retinal endothelial cells. The binding dynamics of Tp0751 to HUVEC via the laminin receptor have been elucidated [207]. The authors subsequently reported that Tp0751 binding to HUVEC monolayers induced rearrangement of the junctional protein VE-cadherin at the cell-cell interface [222]. They observed that Nichols strain *T. pallidum* localised to the cell-cell junctions, and a low, but measurable, percentage were able to transmigrate the endothelial cell monolayer without increasing barrier permeability. This study also reported cholesterol-dependent transcellular transmigration by *T. pallidum*, although the mechanism was not fully elucidated.

A study using the HMEC-1 human dermal microvascular endothelial cell line showed that exposure to live Nichols strain *T. pallidum* triggered phosphorylation of intermediate filament protein, vimentin, by AKT, and resulted in dissociation of the extracellular fibronectin matrix and increased migration of spirochaetes through the endothelial cell monolayer [223]. Binding of *T. pallidum* to HMEC-1 monolayers pre-treated with laminin was increased, but pre-treatment with fibronectin reduced spirochaete transmigration. Similar results were reported in a blood-brain barrier model using HCMEC/D3 human brain microvascular endothelial cell monolayers, which up-regulated the proteoglycan cleavage enzyme, a disintegrin and metalloproteinase with thrombospondin motifs-5, and became more permeable after incubation with live Nichols strain *T. pallidum* [224].

While adherence and transmigration of *T. pallidum* spirochaetes have not yet been investigated in human retinal pigment epithelial cells or retinal vascular endothelial cells, alterations of junctional molecule expression or localisation, cytoskeletal alterations, and degradation of the ECM could be expected to increase transmigration of the inner and outer BRB by *T. pallidum* or responding leucocytes.

### Impact of *Treponema pallidum* on lymphocyte interactions with barrier cells

Although T-cells and B-cells predominate in secondary skin lesions and the CSF of patients with syphilis, no studies have examined the interaction of lymphocytes and cell barriers in the context of ocular syphilis. In the absence of direct evidence, studies using barrier cells derived from other tissue may shed light on how *T. pallidum* influences lymphocyte-barrier cell interactions. Immunohistochemistry for CD4, CD8, and CD20 in tissue sections from 3 brain autopsy specimens from patients with tertiary syphilis demonstrated the presence of T-cells and B-cells in the perivascular infiltrate, along with altered expression of endothelial junctional molecules, suggesting blood-brain barrier breakdown allowed lymphocyte extravasation into the surrounding tissues [117]. Re-stimulation experiments with T-cells isolated from the peripheral blood of patients with syphilis indicated the presence of small populations of circulating *T. pallidum* antigen-specific CD4 + T-cells [225]. Human umbilical vein cells up-regulated CXCL8, CCL20, and tissue factor production when co-cultured with TpF1-specific CD4 + T-cells isolated from patients with tertiary syphilis, molecules that could promote the recruitment of leucocytes [226].

### Influence of *Treponema pallidum* on dendritic cell interactions with barrier cells

Dendritic cells (DCs) are an understudied leucocyte subpopulation in the context of syphilis and ocular syphilis. Studies of human DCs showed that phagocytosis of live *T. pallidum* or exposure to recombinant *T. pallidum* proteins induced DC maturation, characterised by the production of pro-inflammatory cytokines such as TNF, IL-1β, and IL-6, expression of ICAM-1, and up-regulated surface expression of HLA-DR, CD80, and CD86 [227, 228]. An interesting study using exosomes derived from primary human DCs demonstrated that treatment with exosomes from *T. pallidum*-exposed DCs increased NFκB signalling via activation of TLR4 and MYD88 in HUVECs, leading to up-regulated production of IL-1β, IL-6, and TNF [229].

### Impact of *Treponema pallidum* on monocyte and macrophage interactions with barrier cells

Experiments using microvesicles derived from THP-1 monocytes stimulated with Nichols strain *T. pallidum* or recombinant Tp0574 showed increased expression of cell adhesion molecules, ICAM-1 and vascular cell adhesion molecule 1, on microvesicle-exposed HUVECs, leading to increased THP-1 monocyte binding to the HUVEC monolayer [230, 231]. Exposure of human dermal vascular smooth muscle cells to Nichols strain *T. pallidum* also led to activation of NFκB signalling, and increased expression of CCL2 and ICAM-1. Binding and dual chamber migration assays showed increased adhesion of THP-1 monocytes to the smooth muscle cell monolayer and increased migration towards supernatant from *T. pallidum*-stimulated smooth muscle cells, which were reduced by anti-ICAM-1 and anti-CCL2 antibody blockade, respectively [201]. These reports indicate that pro-inflammatory cytokine production by multiple leucocyte subsets exposed to *T. pallidum* could contribute to the pathogenesis of ocular syphilis.

Authors of a recent review suggested TNF, IL-1β, IL-6, CCL2, and CXCL8 as key cytokines mediating endothelial barrier breakdown in non-infectious uveitis, but there are no mechanistic studies examining their role in ocular syphilis specifically [232]. A number of reports have documented macrophage polarisation, inflammasome activation, and production of chemokines and pro-inflammatory cytokines, including CXCL8, TNF, IL-1β, and IL-6, in monocytes and macrophages following exposure to live *T. pallidum* or recombinant *T. pallidum* proteins [233–238]. On the other hand, one study showed that co-culture of *T. pallidum*-stimulated macrophages with HCMEC/D3 human brain microvascular endothelial cells resulted in reduced supernatant concentrations of TNF and IL-1β, and increased IL-6, suggesting barrier cells could modify leucocyte activation during *T. pallidum* infection [239]. Barrier cells, such as retinal pigment epithelial cells, also may play an immunomodulatory role, potentially altering leucocyte activation and down-regulating the inflammatory response.

### Summary

The outer and inner BRBs are critical for maintaining the immune microenvironment of the retina. In ocular syphilis, breakdown of these barriers, with leucocyte recruitment and activation, will promote retinal inflammation in response to *T. pallidum*. Results from historic histopathological studies, and CSF or aqueous analysis from patients suggest a prominent role for monocytes and macrophages in driving the inflammation. Aqueous profiling also reveals high concentrations of numerous pro-inflammatory cytokines. *T. pallidum* spirochaetes adhere to and transmigrate endothelial cell monolayers via interactions with ECM components and host cell receptors, a potential mechanism of retinal invasion.

## Conclusions and future directions

**Fig. 5:**
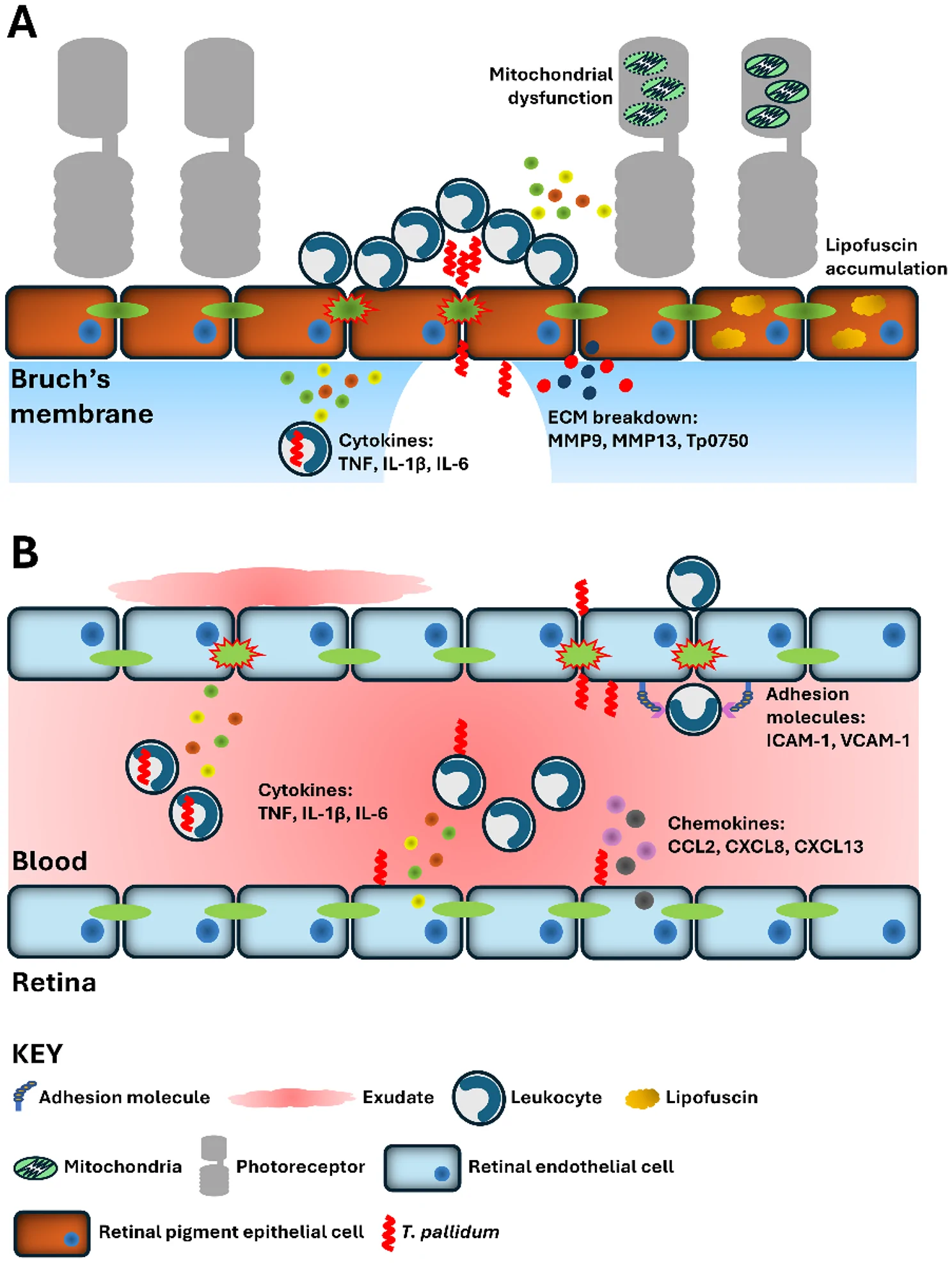
Putative mechanisms of ocular inflammation in syphilis. Cartoon representation of the mechanisms that might occur at the outer (**A**) and inner (**B**) blood-retinal barriers (BRBs) in a patient with ocular syphilis. Extracellular matrix degradation by treponemal proteins and matrix metalloproteinases precedes adhesion of *T. pallidum* to the retinal pigment epithelium and retinal vascular endothelium, triggering expression of adhesion molecules, chemokines, and inflammatory cytokines. Responding leucocytes are activated by locally produced cytokines or *T. pallidum* exposure, amplifying the inflammatory response and exacerbating breakdown of the BRBs. This promotes *T. pallidum* invasion of the retina, leakage from compromised retinal vessels, and leucocyte infiltration. Exposure to *T. pallidum* directly alters junctional protein expression and/or localization in the retinal pigment epithelium and retinal endothelium. Exposure of the retinal pigment epithelium to *T. pallidum* and/or inflammatory cytokines impairs its metabolic and visual cycle functions, and leads to mitochondrial dysfunction in photoreceptors. CCL = c-c motif chemokine ligand; CXCL = c-x-c motif chemokine ligand; ECM = extracellular matrix; ICAM-1 = intercellular adhesion molecule 1; IL = interleukin; MMP = matrix metalloproteinase; Tp = treponemal protein TNF = tumour necrosis factor; VCAM-1 = vascular cell adhesion molecule 1

In vitro models of ocular syphilis are lacking to date, but cellular and molecular changes at the outer and inner BRBs, similar to those observed in non-ocular vascular endothelial and epithelial cells, likely contribute to the retinal inflammation that characterises ocular syphilis. Considering common posterior segment imaging features, the findings in aqueous studies, and the results of in vitro* T. pallidum* infection experiments, Fig. 5 presents a model of the pathogenic mechanisms that may underpin ocular syphilis.

In a landmark 2018 paper, Edmondson et al. reported the first continuous in vitro cultivation of *T. pallidum* [38]. This technique has allowed for expanded in vitro antibiotic resistance and susceptibility testing [240–242] and, for the first time, genetic modification of *T. pallidum* including production of gene knockout strains and fluorescent reporter spirochaetes [243–245].

Tractability to genetic modification raises the possibility of utilising genetic screening tools (e.g., phenotype screening by clustered regularly interspaced short palindromic repeats [CRISPR] interference), used successfully in other pathogenic bacteria [246], to identify those genes most critical for *T. pallidum* survival and pathogenicity. Adaptation of in vitro culture has also been used to isolate *T. pallidum* directly from fresh or frozen clinical samples which, along with whole genome sequencing, may now allow for studies examining the contributions of genomic features to pathogenicity of isolates from patients with ocular syphilis [247, 248].

Extraocular epithelial cells exposed to *T. pallidum* alter gene expression in pathways involved in pro-inflammatory signalling, and vascular smooth muscle cells up-regulate expression of chemokines and adhesion molecules in response to spirochaetes, while also modulating leucocyte behaviour [195, 201]. Soluble mediators released by leucocytes exposed to *T. pallidum* or spirochaetal proteins increase adhesion molecule expression and more avid binding to endothelial cell monolayers [230, 231]. Adaptation of in vitro retinal cell infection models, previously developed to study *Toxoplasma gondii*, dengue virus and Ebolavirus infection, to examine the impact of *T. pallidum* on different cell populations will yield crucial information about the development of retinal inflammation during ocular syphilis [100, 249–251]. Furthermore, as recently described for *T. gondii* infection, retinal organoids may prove useful in studying the effects of *T. pallidum* on Müller cells, photoreceptors, and retinal ganglion cells in a three-dimensional cell culture system [252].

Although *T. pallidum* has no orthologs of classical bacterial virulence factors, attachment and invasion are considered essential to its pathogenicity [253]. Dysregulation of cell-cell junction pathways along with cytoskeletal rearrangement in response to *T. pallidum* have been shown in extraocular epithelial and endothelial cells. Thus, an important step in elucidating the pathogenic mechanisms in ocular syphilis will be determining the molecular interactions mediating *T. pallidum* attachment to retinal pigment epithelial cells and retinal vascular endothelial cells, and the effect of the interaction on the barrier function of those same cells. Attachment and invasion assays using existing retinal endothelial cell and retinal pigment epithelial cell barrier models [101, 254], potentially combined with ablation of candidate *T. pallidum* adhesin genes, will allow for precise dissection of the molecular interactions that precipitate invasion of the retina.

Advances in long-term in vitro culture of *T. pallidum* and recent progress in genetic modification of the bacterium now give us the ability to answer crucial questions about how tissue damage occurs in ocular syphilis. The gaps in knowledge of the disease mechanisms are not merely academic, but represent a major roadblock to achieving better clinical outcomes. While the diagnosis of syphilitic uveitis by serology is straightforward [2], mechanistic research can support identification of oculotropic strains and thereby support public health surveillance. Linking molecules produced in the eye during an infection with vision outcomes in patients could identify biomarkers for a poor prognosis, identifying individuals for intensive treatment and careful follow-up. Understanding the role of the immune response in contributing to ocular damage, and particularly in the outer retina, would point to targeted therapies. For example, vamikibart, an intravitreally injected therapeutic that targets IL-6 [255], might have therapeutic value in ocular syphilis if the limited work implicating this cytokine in the local pathology can be confirmed. This review on the putative mechanisms of inflammation in ocular syphilis may be viewed as a call to action for researchers to focus new effort on this important infectious eye disease.

## Supplementary Information

Below is the link to the electronic supplementary material.

Supplementary Material 1

## Data Availability

No datasets were generated or analysed during this work.

## Abbreviations

ASPPC: Acute syphilitic posterior placoid chorioretinitis
BRB: Blood-retinal barrier
CCL: c-c motif chemokine ligand
CD: Cluster of differentiation
CNS: Central nervous system
CSF: Cerebrospinal fluid
CXCL: c-x-c motif chemokine ligand
DCs: Dendritic cells
ECM: Extracellular matrix
EZ: Ellipsoid zone
FAF: Fundus autofluorescence
G-CSF: Granulocyte colony-stimulating factor
GM-CSF: Granulocyte-macrophage colony-stimulating factor
HCMEC/D3: Human cerebral microvascular endothelial cell d3
HEK293: Human embryonic kidney 293 cell
HIV: Human immunodeficiency virus
HMEC-1: Human microvascular endothelial cell 1
HUVEC: Human umbilical vein endothelial cell
ICAM-1: Intercellular adhesion molecule 1
IFN: Interferon
IL: Interleukin
JAM: Junctional adhesion molecule
LogMAR: Logarithm of the minimum angle of resolution
LPS: Lipopolysaccharide
MMP: Matrix metalloproteinase
MYD88: Myeloid differentiation primary response 88
NFκB: Nuclear factor of kappa light chain enhancer of B cells
OCCL: Occludin
OCT: Optical coherence tomography
PDL1: Programmed cell death 1 ligand 1
SS14: Street strain 14
SNPs: Single-nucleotide polymorphisms
TIMP1: Tissue inhibitor of metalloproteinases 1
TLR: Toll-like receptor
TNF: Tumour necrosis factor
TRAF: TNF-receptor associated factors
VA: Visual acuity
VEGF: Vascular endothelial growth factor
ZO: Zonula occludens
